# Fast Discriminative Stochastic Neighbor Embedding Analysis

**DOI:** 10.1155/2013/106867

**Published:** 2013-06-18

**Authors:** Jianwei Zheng, Hong Qiu, Xinli Xu, Wanliang Wang, Qiongfang Huang

**Affiliations:** School of Computer Science and Technology, Zhejiang University of Technology, Hangzhou 310023, China

## Abstract

Feature is important for many applications in biomedical signal analysis and living system analysis. A fast discriminative stochastic neighbor embedding analysis (FDSNE) method for feature extraction is proposed in this paper by improving the existing DSNE method. The proposed algorithm adopts an alternative probability distribution model constructed based on its *K*-nearest neighbors from the interclass and intraclass samples. Furthermore, FDSNE is extended to nonlinear scenarios using the kernel trick and then kernel-based methods, that is, KFDSNE1 and KFDSNE2. FDSNE, KFDSNE1, and KFDSNE2 are evaluated in three aspects: visualization, recognition, and elapsed time. Experimental results on several datasets show that, compared with DSNE and MSNP, the proposed algorithm not only significantly enhances the computational efficiency but also obtains higher classification accuracy.

## 1. Introduction

In recent years, dimensional reduction which can reduce the curse of dimensionality [1] and remove irrelevant attributes in high-dimensional space plays an increasingly important role in many areas. It promotes the classification, visualization, and compression of the high dimensional data. In machine learning, dimension reduction is used to reduce the dimension by mapping the samples from the high-dimensional space to the low-dimensional space. There are many purposes of studying it: firstly, to reduce the amount of storage, secondly, to remove the influence of noise, thirdly, to understand data distribution easily, and last but not least, to achieve good results in classification or clustering.

Currently, many dimensional reduction methods have been proposed, and they can be classified variously from different perspectives. Based on the nature of the input data, they are broadly categorized into two classes: linear subspace methods which try to find a linear subspace as feature space so as to preserve certain kind of characteristics of observed data, and nonlinear approaches such as kernel-based techniques and geometry-based techniques; from the class labels' perspective, they are divided into supervised learning and unsupervised learning; furthermore, the purpose of the former is to maximize the recognition rate between classes while the latter is for making the minimum of information loss. In addition, judging whether samples utilize local information or global information, we divide them into local method and global method.

We briefly introduce several existing dimensional reduction techniques. In the main linear techniques, principal component analysis (PCA) [2] aims at maximizing the variance of the samples in the low-dimensional representation with a linear mapping matrix. It is global and unsupervised. Different from PCA, linear discriminant analysis (LDA) [3] learns a linear projection with the assistance of class labels. It computes the linear transformation by maximizing the amount of interclass variance relative to the amount of intraclass variance. Based on LDA, marginal fisher analysis (MFA) [4], local fisher discriminant analysis (LFDA) [5], and max-min distance analysis (MMDA) [6] are proposed. All of the three are linear supervised dimensional reduction methods. MFA utilizes the intrinsic graph to characterize the intraclass compactness and uses meanwhile the penalty graph to characterize interclass separability. LFDA introduces the locality to the LFD algorithm and is particularly useful for samples consisting of intraclass separate clusters. MMDA considers maximizing the minimum pairwise samples of interclass.

To deal with nonlinear structural data, which can often be found in biomedical applications [7–10], a number of nonlinear approaches have been developed for dimensional reduction. Among these kernel-based techniques and geometry-based techniques are two hot issues. Kernel-based techniques attempt to obtain the linear structure of nonlinearly distributed data by mapping the original inputs to a high-dimensional feature space. For instance, kernel principal component analysis (kernel PCA) [11] is the extension of PCA using kernel tricks. Geometry-based techniques, in general, are known as manifold learning techniques such as isometric mapping (ISOMAP) [12], locally linear embedding (LLE) [13], Laplacian eigenmap (LE) [14], Hessian LLE (HLLE) [15], and local tangent space alignment (LTSA) [16]. ISOMAP is used for manifold learning by computing the pairwise geodesic distances for input samples and extending multidimensional scaling. LLE exploits the linear reconstructions to discover nonlinear structure in high-dimensional space. LE first constructs an undirected weighted graph, and then recovers the structure of manifold by graph manipulation. HLLE is based on sparse matrix techniques. As for LTSA, it begins by computing the tangent space at every point and then optimizes to find an embedding that aligns the tangent spaces.

Recently, stochastic neighbor embedding (SNE) [17] and extensions thereof have become popular for feature extraction. The basic principle of SNE is to convert pairwise Euclidean distances into probabilities of selecting neighbors to model pairwise similarities. As extension of SNE, *t*-SNE [18] uses Student's *t*-distribution to model pairwise dissimilarities in low-dimensional space and it alleviates the optimization problems and the crowding problem of SNE by the methods below: (1) it uses a symmetrized version of the SNE cost function with simpler gradients that was briefly introduced by Cook et al. [19], and (2) it employs a heavy-tailed distribution in the low-dimensional space. Subsequently, Yang et al. [20] systematically analyze the characteristics of the heavy-tailed distribution and the solutions to crowding problem. More recently, Wu et al. [21] explored how to measure similarity on manifold more accurately and proposed a projection approach called manifold-oriented stochastic neighbor projection (MSNP) for feature extraction based on SNE and *t*-SNE. MSNP employs Cauchy distribution rather than standard Student's *t*-distribution used in *t*-SNE. In addition, for the purpose of learning the similarity on manifold with high accuracy, MSNP uses geodesic distance for characterizing data similarity. Though MSNP has many advantages in terms of feature extraction, there is still a drawback in it: MSNP is an unsupervised method and lacks the idea of class label, so it is not suitable for pattern identification. To overcome the disadvantage of MSNP, we have done some preliminary work and presented a method called discriminative stochastic neighbor embedding analysis (DSNE) [22]. DSNE effectively resolves the problems above, but since it selects all the training samples as their reference points, it has high computational cost and is thus computationally infeasible for the large-scale classification tasks with high-dimensional features [23, 24]. On the basis of our previous research, we present a method called fast discriminative stochastic neighbor embedding analysis (FDSNE) to overcome the disadvantages of DSNE in this paper.

The rest of this paper is organized as follows: in Section 2, we introduce in detail the proposed FDSNE and briefly compare it with MSNP and DSNE in Section 3.  Section 4 gives the nonlinear extension of FDSNE. Furthermore, experiments on various databases are presented in Section 5. Finally, Section 6 concludes this paper and several issues for future works are described.

## 2. Fast Discriminative Stochastic Neighbor Embedding Analysis

Consider a labeled data samples matrix as

(1) $$\begin{array}{c} X=\left\{x_{1}^{1},\ldots ,x_{N_{1}}^{1},x_{1}^{2},\ldots ,x_{N_{2}}^{2},\ldots ,x_{1}^{C},\ldots ,x_{N_{C}}^{C}\right\}, \end{array}$$

where **x**_*i*_^*c*^ ∈ *R* ^*d*^ is a *d*-dimensional sample and means the *i*th sample in the *c*th class. *C* is the number of sample classes, *N*_*c*_ is the number of samples in the *c*th class, and *N* = *N*_1_ + *N*_2_ + ⋯+*N*_*C*_.

In fact, the basic principle of FDSNE is the same as *t*-SNE which is to convert pairwise Euclidean distances into probabilities of selecting neighbors to model pairwise similarities [18]. Since the DSNE selects all the training samples as its reference points, it has high computational cost and is thus computationally infeasible for the large-scale classification tasks with high-dimensional features. So according to the KNN classification rule, we propose an alternative probability distribution function which makes the label of target sample determined by its first *K*-nearest neighbors in FDSNE. In this paper, NH_*l*_(**x**_*i*_) and NM_*l*_(**x**_*i*_) are defined. They, respectively, denote the *l*th-nearest neighbor of **x**_*i*_ from the same class and the different classes in the transformed space. Mathematically, the joint probability *p*_*ij*_ is given by

(2) $$\begin{array}{c} p_{ij}=\{\begin{array}{cc} \frac{\exp\left(-d_{ij}^{2}/2\lambda^{2}\right)}{\sum_{t\in H_{m}}\exp\left(-d_{mt}^{2}/2\lambda^{2}\right)} & \forall j\in H_{i}\\ \frac{\exp\left(-d_{ij}^{2}/2\lambda^{2}\right)}{\sum_{t\in M_{m}}\exp\left(-d_{mt}^{2}/2\lambda^{2}\right)} & \forall j\in M_{i}\\ 0 & \text{otherwise}. \end{array} \end{array}$$

In formula (2), $d_{ij}=||x_{i}-x_{j}||=\sqrt{{\left(x_{i}-x_{j}\right)}^{T}(x_{i}-x_{j})}$ is the Euclidian distance between two samples **x**_*i*_ and **x**_*j*_, the parameter *λ* is the variance parameter of Gaussian which determines the value of *p*_*ij*_,  *H*_*i*_ = {*j* | 1 ≤ *j* ≤ *N*, 1 ≤ *i* ≤ *N*, **x**_*j*_ = NH_*k*_(**x**_*i*_)  and  1 ≤ *k* ≤ *K*_1_}, *H*_*m*_ = {*t* | 1 ≤ *t* ≤ *N*, 1 ≤ *m* ≤ *N*,**x**_*t*_ = NH_*k*_(**x**_*m*_)  and  1 ≤ *k* ≤ *K*_1_}, *M*_*i*_ = {*j* | 1 ≤ *j* ≤ *N*, 1 ≤ *i* ≤ *N*, **x**_*j*_ = NH_*k*_(**x**_*i*_)  and  1 ≤ *k* ≤ *K*_2_}, and *M*_*m*_ = {*t* | 1 ≤ *t* ≤ *N*, 1 ≤ *m* ≤ *N*, **x**_*t*_ = NH_*k*_(**x**_*m*_)  and  1 ≤ *k* ≤ *K*_2_}, and then the denominator in formula (2) means all of the reference points under selection from the same class or the different classes. In particular, the joint probability *p*_*ij*_ not only keeps symmetrical characteristics of the probability distribution matrix but also makes the probability value of interclass data to be 1 and the same for intraclass data.

For low-dimensional representations, FDSNE uses counterparts **y**_*i*_ and **y**_*j*_ of the high-dimensional datapoints **x**_*i*_ and **x**_*j*_. It is possible to compute a similar joint probability via the following expression:

(3) $$\begin{array}{c} q_{ij}=\{\begin{array}{cc} \frac{{\left(1+d_{ij}^{2}\left(A\right)\right)}^{-1}}{\sum_{t\in H_{m}}{\left(1+d_{mt}^{2}\left(A\right)\right)}^{-1}} & \forall j\in H_{i}\\ \frac{{\left(1+d_{ij}^{2}\left(A\right)\right)}^{-1}}{\sum_{t\in M_{m}}{\left(1+d_{mt}^{2}\left(A\right)\right)}^{-1}} & \forall j\in M_{i}\\ 0 & \text{otherwise}. \end{array} \end{array}$$

In what follows, we introduce the transformation by a linear projection: **y**_*i*_ = **A** **x**_*i*_  (**A** ∈ **R** ^*r*×*d*^) so that $d_{ij}(A)=||y_{i}-y_{j}||=||Ax_{i}-Ax_{j}||=\sqrt{{\left(x_{i}-x_{j}\right)}^{T}A^{T}A(x_{i}-x_{j})}$. Then by simple algebra formulation, formula (3) has the following equivalent expression:

(4) qij={(1+(xi−xj)TATA(xi−xj))−1∑t∈Hm(1+(xm−xt)TATA(xm−xt))−1∀j∈Hi(1+(xi−xj)TATA(xi−xj))−1∑t∈Mm(1+(xm−xt)TATA(xm−xt))−1∀j∈Mi0otherwise.

Note that all data have the intrinsic geometry distribution and there is no exception for intraclass samples and interclass samples. Then the same distribution is required to hold in feature space. Since the Kullback-Leiber divergence [25] is wildly used to quantify the proximity of two probability distributions, we choose it to build our penalty function here. Based on the above definition, the function can be formulated as:

(5) $$\begin{array}{c} \min C\left(A\right)=\sum_{\forall j\in H_{i}}p_{ij}\log\frac{p_{ij}}{q_{ij}}+\sum_{\forall j\in M_{i}}p_{ij}\log\frac{p_{ij}}{q_{ij}}. \end{array}$$

In this work, we use the conjugate gradient method to minimize *C*(**A**). In order to make the derivation less cluttered, we first define four auxiliary variables *w*_*ij*_, *u*_*ij*_, *u*_*ij*_^*H*^, and *u*_*ij*_^*M*^ as:

(6) $$\begin{array}{c} w_{ij}={\left[1+{\left(x_{i}-x_{j}\right)}^{T}A^{T}A\left(x_{i}-x_{j}\right)\right]}^{-1},\\ u_{ij}=\left(p_{ij}-q_{ij}\right)w_{ij},\\ u_{ij}^{H}=\{\begin{array}{cc} u_{ij} & \forall j\in H_{i}\\ 0 & \text{otherwise}, \end{array}\\ u_{ij}^{M}=\{\begin{array}{cc} u_{ij} & \forall j\in M_{i}\\ 0 & \text{otherwise}. \end{array} \end{array}$$

Then differentiating *C*(**A**) with respect to the transformation matrix **A** gives the following gradient, which we adopt for learning:

(7) dC(A)d(A)=∑∀j∈Hipijqij(qij)′+∑∀j∈Mipijqij(qij)′=2A[∑∀j∈Hipij(xi−xj)(xi−xj)T1+(xi−xj)TATA(xi−xj)] −2A[∑∀j∈Hipij(∑t∈Hm(1+(xm−xt)TATA(xm−xt))−2            ×(xm−xt)(xm−xt)T)     ×(∑t∈Hm(1+(xm−xt)TATA(xm−xt))−1)−1] +2A[∑∀j∈Mipij(xi−xj)(xi−xj)T1+(xi−xj)TATA(xi−xj)] −2A[∑∀j∈Mipij(∑t∈Mm(1+(xm−xt)TATA(xm−xt))−2             ×(xm−xt)(xm−xt)T)     ×(∑t∈Mm(1+(xm−xt)TATA(xm−xt))−1)−1]=2A[∑∀j∈Hipijwij(xi−xj)(xi−xj)T     −∑t∈Hmqmtwmt(xm−xt)(xm−xt)T] +2A[∑∀j∈Mipijwij(xi−xj)(xi−xj)T     −∑t∈Mmqmtwmt(xm−xt)(xm−xt)T]=2A[∑∀j∈Hiuij(xi−xj)(xi−xj)T    +∑∀j∈Miuij(xi−xj)(xi−xj)T].

Let **U** ^*H*^ be the *N* order matrix with element *u*_*ij*_^*H*^, and let **U** ^*M*^ be the *N* order matrix with element *u*_*ij*_^*M*^. Note that **U** ^*H*^ and **U** ^*M*^ are symmetric matrices; therefore, **D** ^*H*^ can be defined as a diagonal matrix that each entry is column (or row) sum of **U** ^*H*^ and the same for **D** ^*M*^, that is, **D**_*ii*_^*H*^ = ∑_*j*_ **U**_*ij*_^*H*^ and **D**_*ii*_^*M*^ = ∑_*j*_ **U**_*ij*_^*M*^. With this definition, the gradient expression (7) can be reduced to

(8) dC(A)d(A)=2A{∑∀j∈Hiuij(xi−xj)(xi−xj)T   +∑∀j∈Miuij(xi−xj)(xi−xj)T}=2A{(∑∀j∈HiuijxixiT+∑∀j∈HiuijxjxjT    −∑∀j∈HiuijxixjT−∑∀j∈HiuijxjxiT)  +(∑∀j∈MiuijxixiT+∑∀j∈MiuijxjxjT     −∑∀j∈MiuijxixjT−∑∀j∈MiuijxjxiT)}=4A{(XDHXT−XUHXT)  +(XDMXT−XUMXT)}=4A{X(DH−UH+DM−UM)XT}.

Once the gradient is calculated, our optimal problem (5) can be solved by an iterative procedure based on the conjugate gradient method. The description of FDSNE algorithm can be given by the following.

**Step 1**

Collect the sample matrix **X** with class labels, and set *K*-nearest neighborhood parameter *K*_1_, *K*_2_, the variance parameter *λ*, and the maximum iteration times *Mt*.

**Step 2**

Compute the pairwise Euclidian distance for **X** and compute the joint probability *p*_*ij*_ by utilizing formula (2) and class labels.

**Step 3 (set *t* = 1 : *Mt*)**

We search for the solution in loop: firstly, compute the joint probability *q*_*ij*_ by utilizing formula (4); then, compute gradient *dC*(**A**)/*d*(**A**) by utilizing formula (8); finally, update **A** ^*t*^ based on **A** ^*t*−1^ by conjugate gradient operation.

**Step 4**

Judge whether *C* ^*t*^ − *C* ^*t*−1^ < *ε* (in this paper, we take *ε* = 1*e* − 7) converges to a stable solution or *t* reaches the maximum value *Mt*. If these prerequisites are met, Step 5 is performed; otherwise, we repeat Step 3.

**Step 5**

Output **A** = **A** ^*t*^.

Hereafter, we call the proposed method as fast discriminative stochastic neighbor embedding analysis (FDSNE).

## 3. Comparison with MSNP and DSNE

MSNP is derived from SNE and *t*-SNE, and it is a linear method and has nice properties, such as sensitivity to nonlinear manifold structure and convenience for feature extraction. Since the structure of MSNP is closer to that of FDSNE, we briefly compare FDSNE with MSNP and DSNE in this section.

FDSNE, MSNP, and DSNE use different probability distributions to determine the reference points. The difference can be explained in the following aspects.

Firstly, MSNP learns the similarity relationship of the high-dimensional samples by estimating neighborhood distribution based on geodesic distance metric, and the same distribution is required in feature space. Then the linear projection matrix **A** is used to discover the underlying structure of data manifold which is nonlinear. Finally, the Kullback-Leibler divergence objective function is used to keep pairwise similarities in feature space. So the probability distribution function of MSNP and its gradient used for learning are respectively given by

(9) $$\begin{array}{c} p_{ij}=\frac{\exp\left(-D_{ij}^{\text{geo}}/2\right)}{\sum_{k\neq i}\exp\left(-D_{ik}^{\text{geo}}/2\right)},\\ q_{ij}=\frac{{\left[\gamma^{2}+{\left(x_{i}-x_{j}\right)}^{T}A^{T}A\left(x_{i}-x_{j}\right)\right]}^{-1}}{\sum_{k\neq l}{\left[\gamma^{2}+{\left(x_{k}-x_{l}\right)}^{T}A^{T}A(x_{k}-x_{l})\right]}^{-1}},\\ \min C\left(A\right)=\sum_{i,j}p_{ij}\log\frac{p_{ij}}{q_{ij}}, \end{array}$$

where *D*_*ij*_^geo^ is the geodesic distance for **x**_*i*_ and **x**_*j*_ and *γ* is the freedom degree parameter of Cauchy distribution. 

DSNE selects the joint probability to model the pairwise similarities of input samples with class labels. It also introduces the linear projection matrix **A** as MSNP. The cost function is constructed to minimize the intraclass Kullback-Leibler divergence as well as to maximize the interclass KL divergences. Its probability distribution function and gradient are, respectively, given as by

(10) $$\begin{array}{c} p_{ij}=\{\begin{array}{cc} \frac{\exp\left(-{||x_{i}-x_{j}||}^{2}/2\lambda^{2}\right)}{\sum_{c_{k}=c_{l}}\exp\left(-{||x_{k}-x_{l}||}^{2}/2\lambda^{2}\right)} & \text{if}\;\;c_{i}=c_{j}\\ \frac{\exp\left(-{||x_{i}-x_{j}||}^{2}/2\lambda^{2}\right)}{\sum_{c_{k}\neq c_{m}}\exp\left(-{||x_{k}-x_{m}||}^{2}/2\lambda^{2}\right)} & \text{else } \end{array}\\ q_{ij}=\{\begin{array}{cc} \frac{{\left(1+{\left(x_{i}-x_{j}\right)}^{T}A^{T}A\left(x_{i}-x_{j}\right)\right)}^{-1}}{\sum_{c_{k}=c_{l}}{\left(1+{\left(x_{k}-x_{l}\right)}^{T}A^{T}A\left(x_{k}-x_{l}\right)\right)}^{-1}} & \text{if}\;\;c_{i}=c_{j}\\ \frac{{\left(1+{\left(x_{i}-x_{j}\right)}^{T}A^{T}A\left(x_{i}-x_{j}\right)\right)}^{-1}}{\sum_{c_{k}\neq c_{m}}{\left(1+{\left(x_{k}-x_{m}\right)}^{T}A^{T}A\left(x_{k}-x_{m}\right)\right)}^{-1}} & \text{else}, \end{array}\\ \min C\left(A\right)=\sum_{c_{i}=c_{j}}p_{ij}\log\frac{p_{ij}}{q_{ij}}+\sum_{c_{i}\neq c_{k}}p_{ik}\log\frac{{p_{i}}_{k}}{q_{ik}}. \end{array}$$

Note that on the basis of the DSNE, FDSNE makes full use of class label which not only keeps symmetrical characteristics of the probability distribution matrix but also makes the probability value of interclass data and intraclass data to be 1, and it can effectively overcome large interclass confusion degree in the projected subspace.

Secondly, it is obvious that the selection of reference point in MSNP or DSNE is related to all training samples, while FDSNE only uses the first *K*-nearest neighbors of each sample from all classes. In other words, we propose an alternative probability distribution function to determine whether **x**_*i*_ would pick **x**_*j*_ as its reference point or not. Actually, the computation of gradient during the optimization process mainly determines the computational cost of MSNP and DSNE. So their computational complexity can be written as *O*(2*rNd* + *N* ^2^ *d*) in each iteration. Similarly, the computational complexity of FDSNE is *O*(2*rNd* + *KNd*) in each iteration, where *K* = *K*_1_ + *K*_2_. It is obvious that *K* ≪ *N*. Therefore, FDSNE is faster than MSNP and DSNE during each iteration.

## 4. Kernel FDSNE 

As a bridge from linear to nonlinear, kernel method emerged in the early beginning of the 20th century and its applications in pattern recognition can be traced back to 1964. In recent years, kernel method has attracted wide attention and numerous researchers have proposed various theories and approaches based on it.

The principle of kernel method is a mapping of the data from the input space *R* ^*d*^ to a high-dimensional space *F*, which we will refer to as the *feature space*, by nonlinear function. Data processing is then performed in the feature space, and this can be expressed solely in terms of inner product in the feature space. Hence, the nonlinear mapping need not be explicitly constructed but can be specified by defining the form of the inner product in terms of a Mercer kernel function *κ*.

Obviously, FDSNE is a linear feature dimensionality reduction algorithm. So the remainder of this section is devoted to extend FDSNE to a nonlinear scenario using techniques of kernel methods. Let

(11) $$\begin{array}{c} \kappa \left(x_{i},x_{j}\right)=\langle \varphi \left(x_{i}\right),\varphi \left(x_{j}\right)\rangle \end{array}$$

which allows us to compute the value of the inner product in *F* without having to carry out the map.

It should be noted that we use *φ*_*i*_ to denote *φ*(**x**_*i*_) for brevity in the following. Next, we express the transformation **A** with

(12) $$\begin{array}{c} A={\left[\sum_{i=1}^{N}b_{i}^{\left(1\right)}\varphi_{i},\ldots ,\sum_{i=1}^{N}b_{i}^{\left(r\right)}\varphi_{i}\right]}^{T}. \end{array}$$

We define **B** = [*b* ^(1)^,…,*b* ^(*r*)^]^*T*^ and Φ = [*φ*_1_,…,*φ*_*N*_]^*T*^, and then **A** = **B**Φ. Based on above definition, the Euclidian distance between **x**_*i*_ and **x**_*j*_ in the *F* space is

(13) dijF(A)=||A(φi−φj)||=||BΦ(φi−φj)||=||B(Ki−Kj)||=(Ki−Kj)TBTB(Ki−Kj),

where *K*_*i*_ = [*κ*(**x**_1_,**x**_*i*_),…,*κ*(**x**_*N*_,**x**_*i*_)]^*T*^ is a column vector. It is clear that the distance in the kernel embedding space is related to the kernel function and the matrix **B**. 

In this section, we propose two methods to construct the objective function. The first strategy makes **B** parameterize the objective function. Firstly, we replace *d*_*ij*_(**A**) with *d*_*ij*_^*F*^(**A**) in formula (3) so that *p*_*ij*_^1^, *q*_*ij*_^1^ which are defined to be applied in the high dimensional space *F* can be written as

(14) pij1={exp⁡(−(Kii+Kjj−2Kij)/2λ2)∑t∈Hmexp⁡(−(Kmm+Ktt−2Kmt)/2λ2)∀j∈Hiexp⁡(−(Kii+Kjj−2Kij)/2λ2)∑t∈Mmexp⁡(−(Kmm+Ktt−2Kmt)/2λ2)∀j∈Mi0otherwise,qij1={(1+(Ki−Kj)TBTB(Ki−Kj))−1∑t∈Hm(1+(Km−Kt)TBTB(Km−Kt))−1∀j∈Hi(1+(Ki−Kj)TBTB(Ki−Kj))−1∑t∈Mm(1+(Km−Kt)TBTB(Km−Kt))−1∀j∈Mi0otherwise.

Then, we denote *C*(**B**) by modifying *C*(**A**) via substituting **A** with **B** into the regularization term of formula (5). Finally, by the same argument as formula (7), we give the following gradient:

(15) dC(B)d(B)=∑∀j∈Mipij1qij1(qij1)′+∑∀j∈Hipij1qij1(qij1)′=2B[∑∀j∈Hiuij1(Ki−Kj)(Ki−Kj)T   +∑∀j∈Miuij1(Ki−Kj)(Ki−Kj)T].

In order to make formula (15) easy to be comprehended, *w*_*ij*_^1^, *u*_*ij*_^1^, *u*_*ij*_^1*H*^, and *u*_*ij*_^1*M*^ are given by

(16) $$\begin{array}{c} w_{ij}^{1}={\left[1+{\left(K_{i}-K_{j}\right)}^{T}B^{T}B\left(K_{i}-K_{j}\right)\right]}^{-1},\\ u_{ij}^{1}=\left(p_{ij}-q_{ij}\right)w_{ij}^{1},\\ u_{ij}^{1H}=\{\begin{array}{cc} u_{ij}^{1} & \forall j\in H_{i}\\ 0 & \text{otherwise}, \end{array}\\ u_{ij}^{1M}=\{\begin{array}{cc} u_{ij}^{1} & \forall j\in M_{i}\\ 0 & \text{otherwise}. \end{array} \end{array}$$

Meanwhile, the gradient expression (15) can be reduced to

(17) dC(B)d(B)=2B{∑∀j∈Hiuij1(Ki−Kj)(Ki−Kj)T   +∑∀j∈Miuij1(Ki−Kj)(Ki−Kj)T}=4B{(KD1HKT−KU1HKT)  +(KD1MKT−KU1MKT)}=4B{K(D1H−U1H+D1M−U1M)KT},

where **U** ^1*H*^ is the *N* order matrix with element *u*_*ij*_^1*H*^, and **U** ^*M*^ is the *N* order matrix with element *u*_*ij*_^1*M*^. Note that **U** ^1*H*^ and **U** ^1*M*^ are symmetric matrices; therefore, **D** ^1*H*^ can be defined as a diagonal matrix that each entry is column (or row) sum of **U** ^1*H*^ and the same for **D** ^1*M*^, that is, **D**_*ii*_^1*H*^ = ∑_*j*_ **U**_*ij*_^1*H*^ and **D**_*ii*_^1*M*^ = ∑_*j*_ **U**_*ij*_^1*M*^.

For convenience, we name this kernel method as FKDSNE1.

Another strategy is that we let *C* ^*F*^(**A**) be the objective function in the embedding space *F*. So its gradient can be written as

(18) dCF(A)d(A)=∑∀j∈Mipij1qij1(qij1)′+∑∀j∈Hipij1qij1(qij1)′=2[∑∀j∈Hipij1B(Ki−Kj)(φi−φj)T(1+(Ki−Kj)TBTB(Ki−Kj))] −2[∑∀j∈Hipij1(∑t∈Hm(1+(Km−Kt)TBTB(Km−Kt))−2            ×B(Km−Kt)(φm−φt)T)    ×(∑t∈Hm(1+(Km−Kt)TBTB(Km−Kt))−1)−1] +2[∑∀j∈Mipij1B(Ki−Kj)(φi−φj)T1+(Ki−Kj)TBTB(Ki−Kj)] −2[∑∀j∈Mipij1(∑t∈Mm(1+(Km−Kt)TBTB(Km−Kt))−2            ×B(Km−Kt)(φm−φt)T)    ×(∑t∈Mm(1+(Km−Kt)TBTB(Km−Kt))−1)−1]=2[∑∀j∈Hipij1wij1BQij(Ki−Kj)−∑t∈Hmqmt1wmt1BQmt(Km−Kt)]Φ +2[∑∀j∈Mipij1wij1BQij(Ki−Kj)−∑t∈Mmqmt1wmt1BQmt(Km−Kt)]Φ=2[∑∀j∈Hiuij1BQij(Ki−Kj)+∑∀j∈Miuij1BQij(Ki−Kj)]Φ

in this form, *Q*_*ij*_^(*K*_*i*_−*K*_*j*_)^ can be regard as the *N* × *N* matrix with vector *K*_*i*_ − *K*_*j*_ in the *i*th column, and vector *K*_*j*_ − *K*_*i*_ in the *j*th column and the other columns are all zeros.

This method is termed as FKDSNE2. Note that Φ is a constant matrix. Furthermore, the observations of formula (18) make us know that updating the matrix **A** in the optimization only means updating the matrix **B**. Additionally, Φ does not need to be computed explicitly. Therefore, we do not need to explicitly perform the nonlinear map *φ*(**x**) to minimize the objective function *C* ^*F*^(**A**). The computational complexity of FKDSNE1 and FKDSNE2, is respectively, *O*(2*rN* ^2^ + *rN* *K*) and *O*(2*rKN* + *rN* ^2^) in each iteration. Hence, it is obvious that FKDSNE2 is faster than FKDSNE1 during each iteration.

## 5. Experiments

In this section, we evaluate the performance of our FDSNE, FKDSNE1, and FKDSNE2 methods for feature extraction. Three sets of experiments are carried out on Columbia Object Image Library (COIL-20) (http://www1.cs.columbia.edu/CAVE/software/softlib/coil-20.php), US Postal Service (USPS) (http://www.cs.nyu.edu/~roweis/data.html), and ORL (http://www.cam-orl.co.uk) face datasets to demonstrate their good behavior on visualization, accuracy, and elapsed time. In the first set of experiments, we focus on the visualization of the proposed methods which are compared with that of the relevant algorithms, including SNE [17], *t*-SNE [18], and MSNP [21]. In the second set of experiments, we apply our methods to recognition task to verify their feature extraction capability and compare them with MSNP and DSNE [22]. Moreover, the elapsed time of FDSNE, FKDSNE1, FKDSNE2, and DSNE is compared in the third set of experiments. In particular, the Gaussian RBF kernel *κ*(**x**, **x**′) = exp⁡⁡(−||**x**−**x**′||^2^/2*σ* ^2^) is chosen as the kernel function of FKDSNE1 and FKDSNE2, where *σ* is set as the variance of the training sample set of **X**.

### 5.1. COIL-20, USPS, and ORL Datasets

The datasets used in our experiments are summarized as follows.

**Figure 1:**
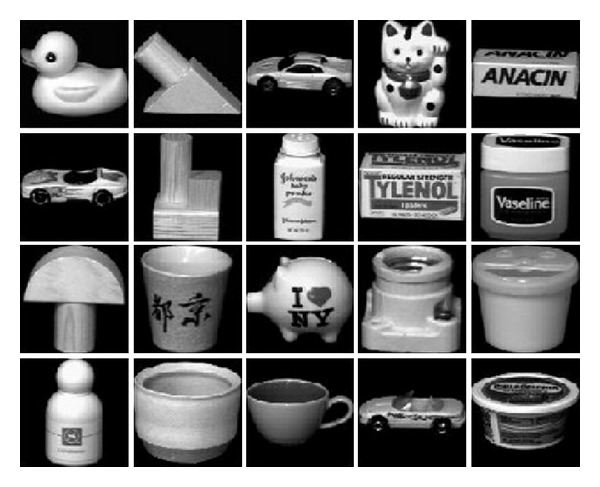
Sample images from COIL-20 dataset.

COIL-20 is a dataset of gray-scale images of 20 objects. The images of each object were taken 5 degrees apart as the object is rotated on a turntable and each object has 72 images. The size of each image is 40 × 40 pixels.  Figure 1 shows sample images from COIL-20 images dataset.

**Figure 2:**
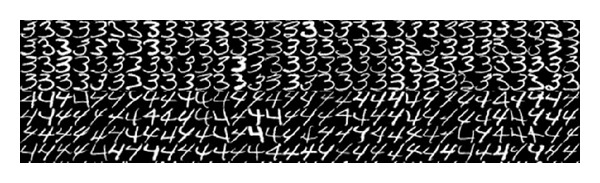
Samples of the cropped images from USPS dataset.

USPS handwritten digit dataset includes 10 digit characters and 1100 samples in total. The original data format is of 16 × 16 pixels.  Figure 2 shows samples of the cropped images from USPS handwritten digits dataset.

**Figure 3:**
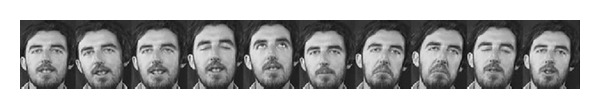
Sample face images from ORL dataset.

ORL consists of gray images of faces from 40 distinct subjects, with 10 pictures for each subject. For every subject, the images were taken with varied lighting condition and different facial expressions. The original size of each image is 112 × 92 pixels, with 256 gray levels per pixel.  Figure 3 illustrates a sample subject of ORL dataset.

### 5.2. Visualization Using FDSNE, FKDSNE1, and FKDSNE2

We apply FDSNE, FKDSNE1, and FKDSNE2 to visualization task to evaluate their capability of classification performance. The experiments are carried out, respectively, on COIL-20, USPS, and ORL datasets. For the sake of computational efficiency as well as noise filtering, we first adjust the size of each image to 32 × 32 pixels on ORL, and then we select five samples from each class on COIL-20, fourteen samples from each class on USPS, and five samples from each class on ORL.

The experimental procedure is to extract a 20-dimensional feature for each image by FDSNE, FKDSNE1, and FKDSNE2, respectively. Then to evaluate the quality of features through visual presentation of the first two-dimensional feature. 

FDSNE, FKDSNE1, and FKDSNE2 are compared with three well known visualization methods for detecting classification performance: (1) SNE, (2) *t*-SNE, and (3) MSPN. The parameters are set as follows: the *K*-nearest neighborhood parameter of FDSNE, FKDSNE1, and FKDSNE2 methods is *K*_1_ = *h* − 1  (let *h* denote the number of training samples in each class), *K*_2_ = 40; for SNE and *t*-SNE, the perplexity parameter is perp = 20 and the iteration number is *Mt* = 1000; for MSNP, the degree freedom of Cauchy distribution is *γ* = 4 and the iteration number is 1000 as well.

**Figure 4:**
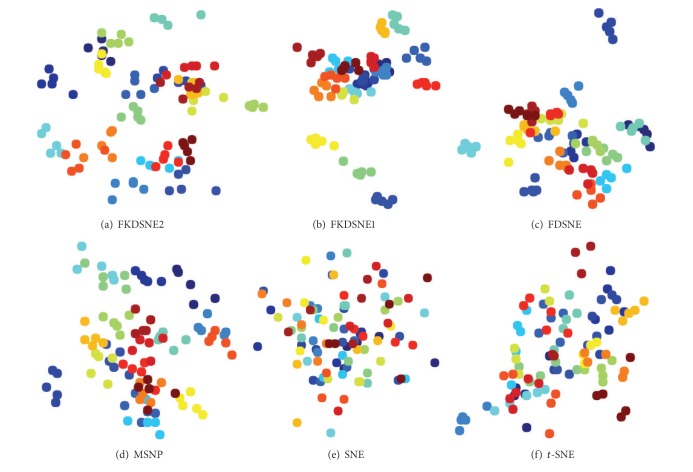
Visualization of 100 images from COIL-20 images dataset.

**Figure 5:**
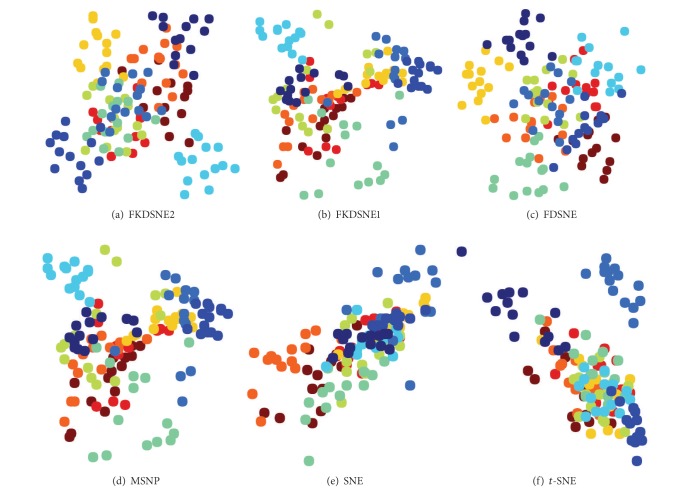
Visualization of 140 images from USPS handwritten digits dataset.

**Figure 6:**
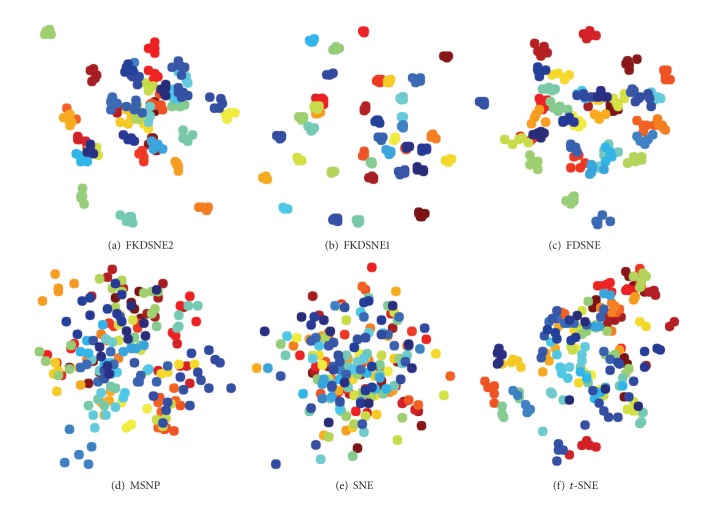
Visualization of 200 face images from ORL faces dataset.

Figures 4, 5, and 6 show the visual presentation results of FDSNE, FKDSNE1, FKDSNE2, SNE, *t*-SNE, and MSNP, respectively, on COIL-20, USPS, and ORL datasets. The visual presentation is represented as a scatterplot in which a different color determines different class information. The figures reveal that the three nearest-neighbor-based methods, that is, FDSNE, FKDSNE1, and FKDSNE2, give considerably better classification result than SNE, *t*-SNE, and MSNP on all datasets, for the separation between classes is quite obvious. In particular, SNE and *t*-SNE not only get less separation for the interclass data but also produce larger intraclass scatter. For MSNP, it has smaller intraclass scatter, but there exists an overlapping phenomenon among classes. With regard to FDSNE, FKDSNE1, and FKDSNE2, we can find from the figures that FKDSNE1 shows the best classification performance among all the algorithms on ORL face dataset, while not on the other two datasets COIL-20 and USPS; thereinto, the classification performance of FKDSNE1 is inferior to FDSNE on COIL-20 while on USPS it is inferior to FKDSNE2. In addition, the clustering qualities and separation degree of FKDSNE1 and FKDSNE2 are obviously better than that of FDSNE.

### 5.3. Recognition Using FDSNE, FKDSNE1, and FKDSNE2

In this subsection, we apply FDSNE, FKDSNE1, and FKDSNE2 to recognition task to verify their feature extraction capability. Nonlinear dimensional reduction algorithms such as SNE and *t*-SNE lack explicit projection matrix for the out-of-sample data, which means they are not suitable for recognition. So we compare the proposed methods with DSNE and MSNP, both of them are linear methods and were proved to be better than existing feature extraction algorithms such as SNE, *t*-SNE, LLTSA, LPP, and so on in [21, 22]. The procedure of recognition is described as follows: firstly, divide dataset into training sample set **X**_train_ and testing sample set **X**_test_ randomly; secondly, the training process for the optimal matrix **A** or **B** is taken for FDSNE, FKDSNE1 and FKDSNE2; thirdly, feature extraction is accomplished for all samples using **A** or **B**; finally, a testing image is identified by a nearest neighbor classifier. The parameters are set as follows: the *K*-nearest neighborhood parameter *K*_1_, *K*_2_  in FDSNE, FKDSNE1, and FKDSNE2 is *K*_1_ = *h* − 1, *K*_2_ = 40; for DSNE, the perplexity parameter is *λ* = 0.1 and the iteration number is *Mt* = 1000; for MSNP, the freedom degree *γ* of Cauchy distribution in MSNP is determined by cross validation and the iteration number is 1000 as well. 

**Figure 7:**
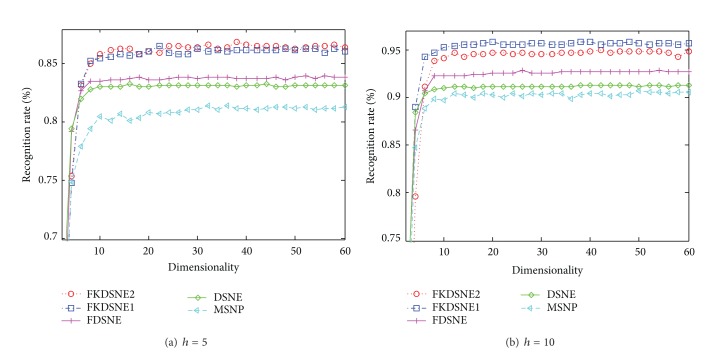
Recognition rate (%) versus subspace dimension on COIL-20.

**Figure 8:**
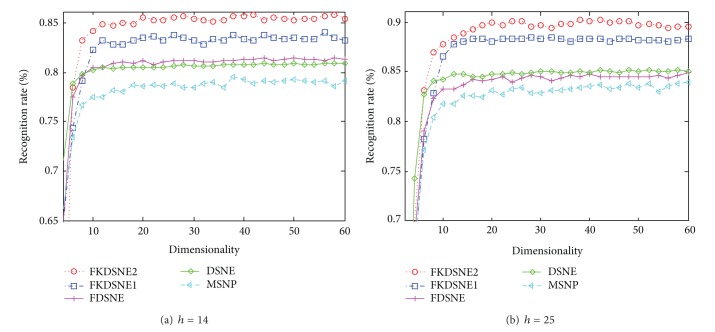
Recognition rate (%) versus subspace dimension on USPS.

**Figure 9:**
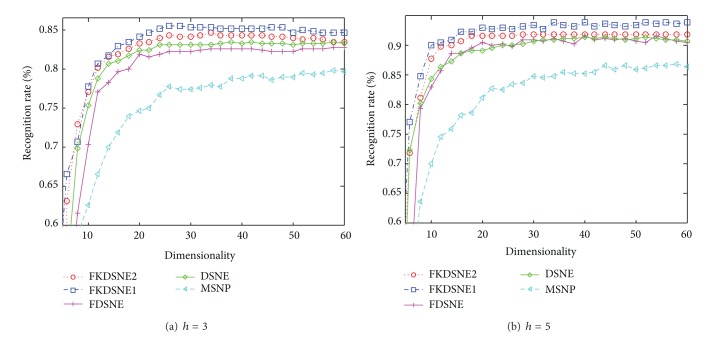
Recognition rate (%) versus subspace dimension on ORL.

Figure 7 demonstrates the effectiveness of different subspace dimensions for COIL-20 ((a): *h* = 5, (b): *h* = 10).  Figure 8 is the result of the experiment in USPS ((a): *h* = 14, (b): *h* = 25), and Figure 9 shows the recognition rate versus subspace dimension on ORL ((a): *h* = 3, (b): *h* = 5). The maximal recognition rate of each method and the corresponding dimension are given in Table 1, where the number in bold stands for the highest recognition rate. From Table 1, we can find that FKDSNE1 and FKDSNE2 outperform MSNP, DSNE, and FDSNE on COIL-20, USPS, and ORL. As can be seen, FKDSNE1 and FKDSNE2 enhance the maximal recognition rate for at least 2% compared with other three methods. Besides, FKDSNE1 and FKDSNE2 achieve considerable recognition accuracy when feature dimension is 20 on the three datasets. It indicates that FKDSNE1 and FKDSNE2 grasp the key character of face images relative to identification with a few features. Though the maximal recognition rate of DSNE and FDSNE is closer to that of FKDSNE1 and FKDSNE2 on ORL dataset, the corresponding dimension of FKDSNE1 and FKDSNE2 is 20 while that of DSNE and FDSNE exceeds 30. From the essence of dimensional reduction, this result demonstrates that FDSNE and DSNE are inferior to FKDSNE1 and FKDSNE2.

### 5.4. Analysis of Elapsed Time

**Figure 10:**
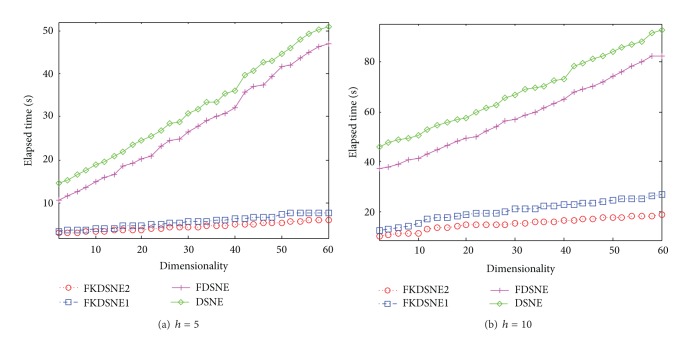
Elapsed time (seconds) versus subspace dimension on COIL-20.

**Figure 11:**
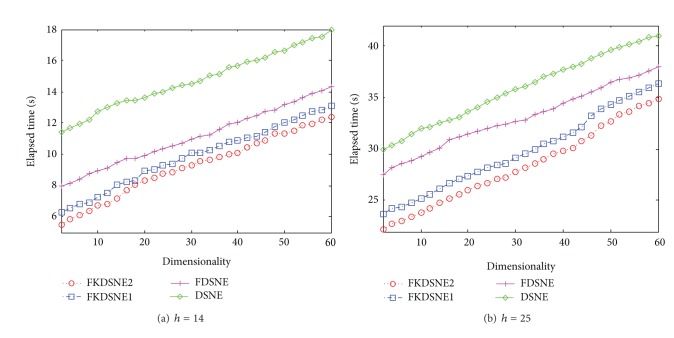
Elapsed time (seconds) versus subspace dimension on USPS.

**Figure 12:**
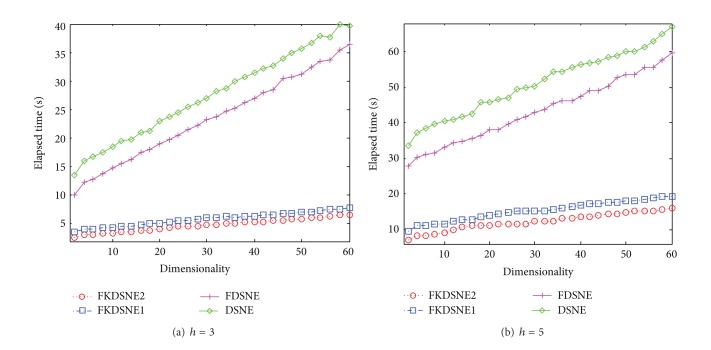
Elapsed time (seconds) versus subspace dimension on ORL.

In this subsection, we further compare the computational efficiency of DSNE, FKDSNE, FKDSNE1, and FKDSNE2. The algorithm MSPN is not compared since its recognition rate is obviously worse than other algorithms. The parameters of the experiment are the same to Section 5.3. Figures 10, 11, and 12, respectively, show the elapsed time of four algorithms under different subspace dimensions on the three datasets. It can be observed from the figures that FKDSNE2 has the lowest computational cost among the four algorithms while DSNE is much inferior to other nearest-neighbor-based algorithms on all datasets. Particularly, on the COIL-20 dataset, the elapsed time of FKDSNE2 is more than 2 times faster than DSNE. As for DSNE and FDSNE, the former is obviously slower than the latter. Besides, for the two kernel methods, FKDSNE2 is notably faster than FKDSNE1, which confirms our discussion in Section 4.

**Figure 13:**
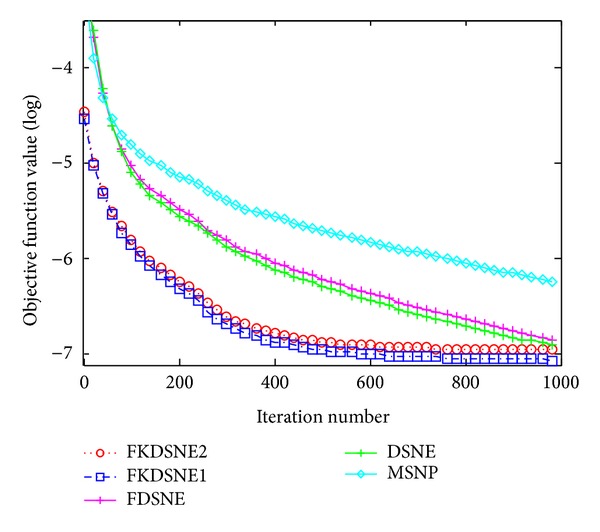
Objective function value (log) versus iterative number on ORL dataset.

Furthermore, kernel-based algorithms FKDSNE1 and FKDSNE2 can effectively indicate the linear structure on high-dimensional space. Their objective function can achieve better values on desirable dimensions. For instance, Figure 13 illustrates the objective function value of MSNP, DSNE, FKDSNE, FKDSNE1, and FKDSNE2 versus iterative number on ORL dataset. It can be found that FKDSNE2 and FKDSNE1 is close to the convergence value 1*e* − 7 while FDSNE and DSNE only achieve 1*e* − 6 and MSNP achieves 1*e* − 5.4 when the iterative number is 400. It means that FKDSNE1 and FKDSNE2 can get the more precise objective function value with less iterative number compared with DSNE and FDSNE; that is to say that, FKDSNE1 and FKDSNE2 can achieve the same value by using forty percent of the elapsed time of DSNE and FDSNE.

## 6. Conclusion

On the basis of DSNE, we present a method called fast discriminative stochastic neighbor embedding analysis (FDSNE) which chooses the reference points in *K*-nearest neighbors of the target sample from the same class and the different classes instead of the total training samples and thus has much lower computational complexity than that of DSNE. Furthermore, since FDSNE is a linear feature dimensionality reduction algorithm, we extend FDSNE to a nonlinear scenario using techniques of kernel trick and present two kernel-based methods: FKDSNE1 and FKDSNE2. Experimental results on COIL-20, USPS, and ORL datasets show the superior performance of the proposed methods. Our future work might include further empirical studies on the learning speed and robustness of FDSNE by using more extensive, especially large-scale, experiments. It also remains important to investigate acceleration techniques in both initialization and long-run stages of the learning.

## Figures and Tables

**Table 1:** The maximal recognition rates (%) versus the subspace dimension.

	COIL-20 *h* = 5	COIL-20 *h* = 10	USPS *h* = 14	USPS *h* = 25	ORL *h* = 3	ORL *h* = 5
MSNP	0.8149 (32)	0.9063 (50)	0.7958 (38)	0.8395 (58)	0.7989 (59)	0.8690 (58)
DSNE	0.8325 (36)	0.9130 (54)	0.8093 (50)	0.8522 (42)	0.8357 (42)	0.9150 (39)
FDSNE	0.8396 (52)	0.9277 (54)	0.8150 (58)	0.8489 (59)	0.8279 (58)	0.9160 (39)
FKDSNE1	0.8651 (22)	**0.9575** (20)	0.8409 (26)	0.8848 (26)	**0.8550** (26)	**0.9405** (24)
FKDSNE2	**0.8689** (28)	0.9491 (22)	**0.8585** (22)	**0.9021** (28)	0.8470 (24)	0.9193 (20)
